# Elevation of S100A4 Expression in Buccal Mucosal Fibroblasts by Arecoline: Involvement in the Pathogenesis of Oral Submucous Fibrosis

**DOI:** 10.1371/journal.pone.0055122

**Published:** 2013-01-31

**Authors:** Cheng-Chia Yu, Chung-Hung Tsai, Hsin-I Hsu, Yu-Chao Chang

**Affiliations:** 1 Institute of Oral Science, Chung Shan Medical University, Taichung, Taiwan; 2 School of Dentistry, Chung Shan Medical University, Taichung, Taiwan; 3 Department of Dentistry, Chung Shan Medical University Hospital, Taichung, Taiwan; 4 Department of Pathology, Chung Shan Medical University Hospital, Taichung, Taiwan; 5 Institute of Medicine, Chung Shan Medical University, Taichung, Taiwan; University of South Florida College of Medicine, United States of America

## Abstract

**Background:**

S100A4, a member of the calcium-binding proteins, is dramatically elevated in a variety of fibrotic diseases. Areca quid chewing is the most important etiological factor in the pathogenesis of oral submucous fibrosis (OSF). OSF has been considered as a pre-cancerous condition of oral mucosa. The aim of this study was to determine the critical role of S100A4 expression in the pathogenesis of OSF both *in vitro* and *in vivo*.

**Methodology/Principal Finding:**

Thirty OSF tissues from areca quid chewers and ten normal buccal mucosa samples without areca quid chewing were analyzed by using immunohistochemistry for S100A4 expression *in vivo*. Collagen gel contraction capability and expression of tissue inhibitor of metalloproteinases 1 (TIMP1)/MMP9 in arecoline-stimulated BMFs with S100A4 knockdown was presented *in vitro*. Initially, S100A4 expression was higher in areca quid chewing-associated OSF specimens than normal buccal mucosa specimens (*p* = 0.001). Arecoline, a major areca nut alkaloid, led to dose- and time-dependent elevation of S100A4 expression in normal buccal mucosa fibroblasts BMFs (*p*<0.05). The additions of pharmacological agents rapamycin (mTOR inhibitor), PD98059 (ERK inhibitor), and Bay117082 (NF-κB inhibitor) were found to inhibit arecoline-induced S100A4 expression (*p*<0.05) in BMFs. Down-regulation of S100A4 by lentiviral infection significantly reversed arecoline-induced collagen gel contraction and TIMP1/MMP9 expression.

**Conclusion/Significance:**

These results suggest that S100A4 expression is significantly up-regulated in OSF specimens. Arecoline-induced S100A4 expression was down-regulated by rapamycin, PD98059, and Bay117082. Targeting S100A4 might be a potential therapeutic target for OSF through TIMP1/MMP9 down-regulation.

## Introduction

Oral submucous fibrosis (OSF) is a chronic progressive scarring disease which characterized by the submucosal accumulation of dense fibrous connective tissue with inflammatory cell infiltration and epithelial atrophy and has been considered as a pre-cancerous condition of oral mucosa [1]. The fibro-elastic changes are almost entirely due to abnormal accumulation of collagen in subepithelial layers, resulting in dense fibrous bands in the mouth [2]. A number of epidemiological evidences, case-series reports, large sized cross sectional surveys, case-control studies, cohort and intervention studies provide over whelming evidences that areca quid chewing is the main etiological factor for the development of OSF [3].

Arecoline, a major areca nut alkaloid, was found to stimulate human buccal mucosa fibroblasts (BMFs) proliferation and collagen synthesis *in vitro* [4]. Up-regulation of vimentin [5], cyclooxygenase-2 [6], tissue inhibitor metalloproteinase-1 (TIMP-1) [7], plasminogen activator inhibitor-1 [8], interleukin-6 [9], keratinocyte growth factor-1 [10], insulin-like growth factor-1, nuclear factor-kappa B (NF-κB) [11], cystatin C [12], and heme oxygenase-1 [13] may contribute to the extracellular matrix (ECM) accumulation in OSF. However, the molecular pathologic mechanism(s) of OSF still remain to be further clarified.

S100A4, a member belongs to S100 super family of calcium-binding proteins (CPB), is associated with the onset and progression of fibrosis in many human tissues, such as liver fibrosis, kidney fibrosis, pulmonary fibrosisc, and cardiac fibrosis [6], [14]–[16]. S100A4 expression is consistently and dramatically upregulated in carbon tetrachloride (CCl_4_)-induced hepatic fibrosis and functions as a marker of primary biliary cirrhosis [14]. Up-regulation of S100A4-positive cells is associated with kidney fibrosis [15]. TGF-β, an inducer of endothelial-mesenchymal transition, was found to induce S100A4 expression in cardiac fibrosis mouse model [6]. Up to now, the role of S100A4 mediated molecular mechanisms in the pathogenesis of OSF is still unclear.

The purpose of this study was to test whether S100A4 expression in OSF and to further explore possible pathogenic mechanisms that may lead to enhanced expression of this molecule in vivo. Moreover, we set out to explore whether expression of S100A4 could be triggered in primary buccal mucosal fibroblasts (BMFs) by arecoline in vitro. In addition, mTOR inhibitor rapamycin, extracellular signal-regulated protein kinase (ERK) inhibitor PD98059, and NF-κB inhibitor bay117082 were added to search the possible signal transduction pathways of arecoline-induced S100A4 expression. Ultimately, we demonstrated the significance of S100A4-mediated signaling on OSF process by measuring collagen gel contraction capability and TIMP1/MMP9 expression of arecoline-stimulated BMFs with S100A4 knockdown in vitro.

## Materials and Methods

### Sample Collection and Immunohistochemistry

Formalin-fixed, paraffin-embedded specimens of ten normal buccal mucosa from non-areca quid chewers, and 30 OSF specimens from areca quid chewers, were drawn from the files of the Department of Pathology, Chung Shan Medical University Hospital. Diagnosis was based on histological examination of hematoxylinand eosin-stained sections. Institutional Review Board permission at the Chung Shan Medical University Hospital was obtained for the use of discarded human tissues (CSMUH No: CSI0249). After deparaffinization and rehydration, the 5-µm tissue sections were processed with antigen retrieval by 1X Trilogy diluted in H2O and heat. The slides were immersed in 3% H2O2 for 10 minutes and washed with PBS 3 times. The tissue sections were then blocked with serum (Vestastain Elite ABC kit, Vector Laboratories, Burlingame, CA) for 30 min, and followed by incubating with the primary antibody and anti-S100A4 (code no. A5114; Dako, Glostrup, Denmark) in phosphate buffer saline (PBS) solution at room temperature for 2 h in a container. Tissue slides were washed with PBS and incubated with biotin-labeled secondary antibody for 30 min and then incubated with streptavidin-horse radish peroxidase conjugates for 30 min and washed with PBS 3 times. Afterwards, the tissue sections were immersed with chromogen 3-3′-diaminobenzidine plus H2O2 substrate solution (Vector® DBA/Ni substrate kit, SK-4100, Vector Laboratories, Burlingame, CA) for 10 min. Hematoxylin was applied for counter-staining (Sigma Chemical Co., USA). Finally, the tumor sections were mounted with a cover slide with Gurr® (BDH Laboratory Supplies, UK) and examined under a microscope. Pathologists scoring the immunohistochemistry were blinded to the clinical data. The interpretation was done in five high-power views for each slide, and 100 cells per view were counted for analysis.

### Reagents

Arecoline was purchased from Sigma (St Louis, MO, USA). Rapamycin, PD98059, or Bay117082 were obtained from Merck (Merck Biosciences, Darmstadt, Germany). All pharmacologic agents were first dissolved in dimethyl sulfoxide and then diluted with the culture medium. The final concentration of solvent in the medium did not exceed 0.25% (v/v). At these concentrations the solvents used were not cytotoxic to BMFs. The final concentrations of rapamycin, PD98059, or Bay117082 used in this study were 100 nM, 10 µM, and 1 µM, respectively.

### Cell Cultivation of BMFs

BMFs were cultured by using an explant technique as described previously [5]. Two healthy individuals were selected from the crown lengthening procedure for this study. The normal buccal mucosa tissue samples were minced using sterile techniques and washed twice in PBS supplemented with antibiotics (100 U/ml penicillin, 100 µg/ml streptomycin and 0.25 µg/ml of fungizone). Explants were placed into 60 mm Petri dishes and maintained in Dulbecco’s modified Eagle’s medium (DMEM) (Gibco Laboratories, Grand Island, NY, USA) supplemented with 10% fetal calf serum (FCS) (Gibco Laboratories, Grand Island, NY, USA) and antibiotics as described above. Cell cultures between the third and eighth passages were used in this study.

### Quantitative Real-time Reverse-transcriptase (RT)-PCR

Total RNA was prepared from cells or tissues using Trizol reagent according to the manufacturer’s protocol (Invitrogen, Carlsbad, CA). qRT-PCRs of mRNAs were reverse-transcribed using the Superscript III first-strand synthesis system for RT-PCR (Invitrogen, Carlsbad, CA). qRT-PCR reactions on resulting cDNAs were performed on an ABI StepOne™ Real-Time PCR Systems (Applied Biosystems, Foster City, CA). Primer sequences are listed in **Table 1**.

**Table 1:** The sequences of the primers for quantitative RT-PCR.

Gene (Accession No.)	Primer Sequence (5′ to 3′)	Product size (bp)	Tm (°C)
S100A4 (NM_002961)	F: GAGCTGCCCAGCTTCTTG	124	59
	R: TGCAGGACAGGAAGACACAG		
TIMP1 (NM_003254)	F: CCCTAAACTCTGCCGTCTCC	159	60
	R: AGTGAGTTGCGGGGTTATGG		
MMP9 (NM_004994)	F: AACCAATCTCACCGACAGGC	154	60
	R: CAGATACGCCCATCACCACC		
GAPDH (NM_003380)	F: CTCATGACCACAGTCCATGC	155	53
	R: TTCAGCTCTGGGATGACCTT		

### Effect of Arecoline on S100A4 Expression in BMFs by Western Blot

Cells arrested in G0 by serum deprivation (0.5% FCS; 48 h) were used in the experiments. Nearly confluent monolayers of BMFs were washed with serum-free Dulbecco’s modified Eagle’s medium and immediately thereafter exposed to various concentrations (0, 5, 15, and 20 µg/mL) of arecoline after 24 h incubation period. Cells were solubilized with sodium dodecyl sulfate-solubilization buffer (5 mM EDTA, 1 mM MgCl2, 50 mM Tris–HCl, pH 7.5 and 0.5% Trition X-100, 2 mM phenylmethylsulfonyl fluoride, and 1 mM *N*-ethylmaleimide) for 30 min on ice. Then, cell lysates were centrifuged at 12,000 *g* at 4°C and the protein concentrations determined with Bradford reagent using bovine serum albumin as standards. Equivalent amounts of total protein per sample of cell extracts were run on a 10% sodium dodecyl sulfate-polyacrylamide gel electrophoresis and immediately transferred to nitrocellulose membranes. The membranes were blocked with phosphate-buffered saline containing 3% bovine serum albumin for 2 h, rinsed, and then incubated with primary antibodies anti-S100A4 (1∶500) in phosphate-buffered saline containing 0.05% Tween 20 for 2 h. After three washes with Tween 20 for 10 min, the membranes were incubated for 1 h with biotinylated secondary antibody diluted 1∶1000 in the same buffer, washed again as described above and treated with 1∶1000 streptavidin-peroxidase solution for 30 min. After a series of washing steps, protein expression was detected by chemiluminescence using an ECL detection kit (Amersham Biosciences UK Limited, England), and relative photographic density was quantitated by scanning the photographic negatives on a gel documentation and analysis system (AlphaImager 2000, Alpha Innotech Corp., San Leandro, CA, USA). Each densitometric value was expressed as the mean ± standard deviation (SD).

### S100A4 Knockdown in Arecoline-treated BMF Cells by Lentiviral-mediated shRNAi

The pLV-RNAi vector was purchased from Biosettia Inc. (Biosettia, San Diego, CA, USA). The method of cloning the double-stranded shRNA sequence is described in the manufacturer’s protocol. Lentiviral vectors expressing short hairpin RNA (shRNA) that targets human *S100A4* (oligonucleotide sequence: Sh-S100A4-1:5′-AAAAGGTGTCCACCTTCCACAAGTATTGGATCCAATACTTGTGGAAGGTGGACACC-3′;Sh-S100A4-2:5′-AAAAGAAGCTGATGAGCAACTTGGATTGGATCCAATCCAAGTTGCTCATCAGCTTC-3′) were synthesized and cloned into pLVRNAi to generate a lentiviral expression vector. Lentivirus production was performed by transfection of plasmid DNA mixture with lentivector plus helper plasmids (VSVG and Gag-Pol) into 293T cells using Lipofectamine 2000 (Invitrogen, Calsbad, CA, USA). Supernatants were collected 48 h after transfection and then were filtered; the viral titers were then determined by FACS at 48 h post-transduction. Subconfluent cells were infected with lentivirus in the presence of 8 µg/ml polybrene (Sigma-Aldrich, St. Louis, Missouri, USA). The red fluorescence protein (RFP), which was co-expressed in lentiviral-infected cells, was served as a selection marker to indicate the successfully infected cells.

### Collagen Gel Contraction Assays

The bioactivity of myofibroblast function was performed by collagen contraction assay kit (Cell BioLabs, Inc., San Diego, CA, USA). 2×10^5^ cells/ml was mixed with cold collagen solution at ratio of 1∶4. Cell/collagen mixture was loaded into 24-well-plate as 0.5 ml/well and covered with 1 ml of cell culture medium after polymerization of collagen. To initiate contraction, collagen gels were gently released from the sides of the culture dishes with a sterile spatula. The changes of collagen gel size (contraction index) were pictured at various times and quantified by IamgeJ software.

### Statistical Analysis

Statistical package of social sciences software (version 13.0) (SPSS, Inc., Chicago, IL, USA) was used for statistical analysis. Student’s *t* test was used to determine statistical significance of the differences between control group and experimental groups; *p* values less than 0.05 were considered statistically significant. The level of statistical significance was set at 0.05 for all tests.

## Results

### S100A4 Significantly Up-regulated in OSF Specimens

**Figure 1:**
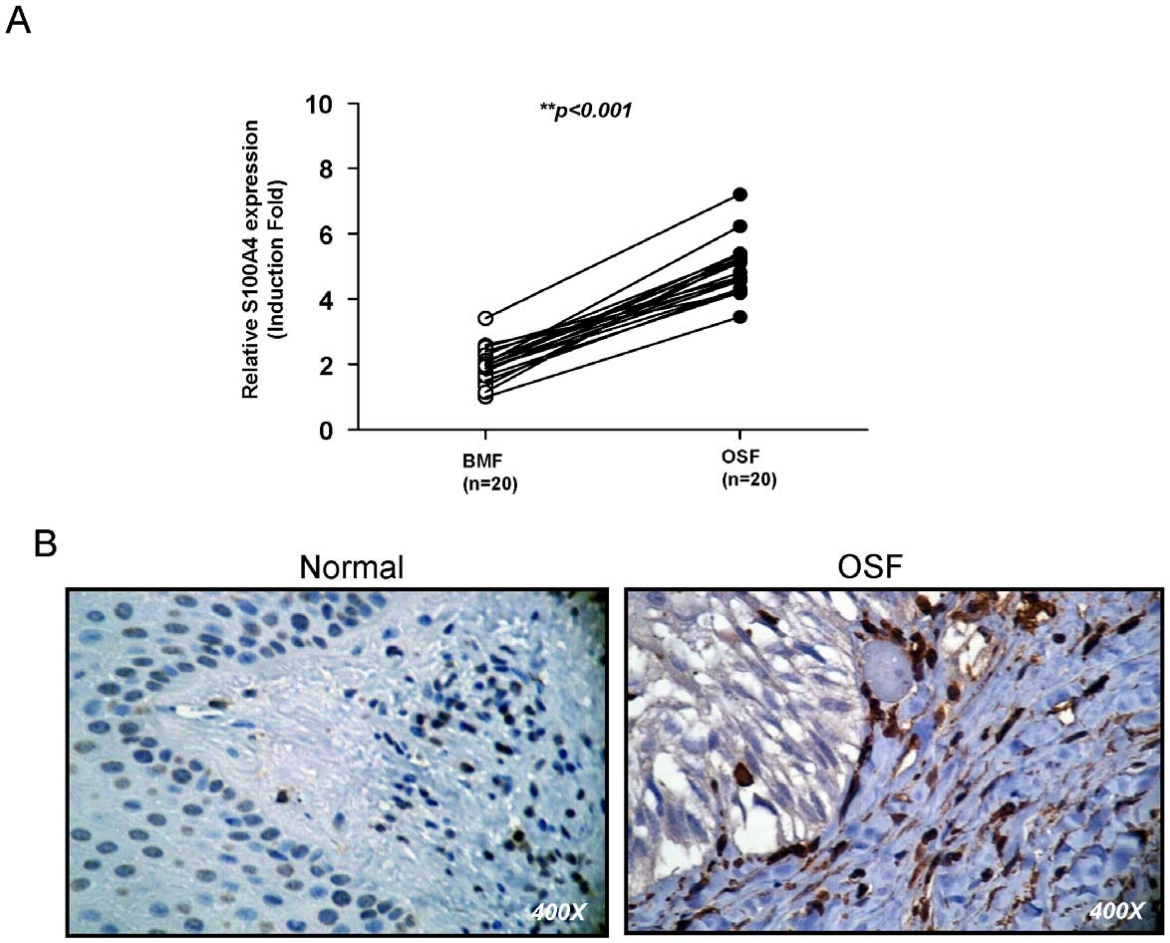
Expression patterns of S100A4 in normal buccal mucosa and OSF specimens. (A) Analysis of S100A4 transcript expression in independent pairs (n = 20) of normal buccal mucosa and OSF specimens by real-time RT-PCR analysis. (B) The representative image of S100A4 expression in normal buccal mucosa and OSF specimens by immunohistochemistry.

To validate the significance of S100A4 in clinical specimens, we collected paired samples of normal buccal mucosa and fibrotic buccal mucosa from OSF patients for real-time RT-PCR analysis. As shown in figure 1A, the levels of S100A4 transcript were higher in OSF than normal specimens. In line with real-time RT-PCR, S100A4 staining was stronger in areca quid chewing-associated OSF specimens than normal specimens (Fig. 1B). Normal buccal mucosa tissues demonstrated very faint S100A4 expression. Differences in S100A4 expression between normal buccal mucosa and OSF were subsequently analyzed using Fisher’s exact test (Table 2). There was a significantly greater S100A4 expression noted in OSF compared to normal buccal mucosa (p = 0.001).

**Table 2:** Expression of S100A4 in normal buccal mucosa and OSF specimens by immunohistochemistry.

	Cases	Weak	Strong
**Normal buccal mucosa**	10	8	2
**OSF**	30	5	25

Statistical analysis was evaluated by Fisher’s exact test.

*** P = 0.001.

### Arecoline Increased S100A4 Expression in a Dose- and Time-dependent Manner in BMFs

**Figure 2:**
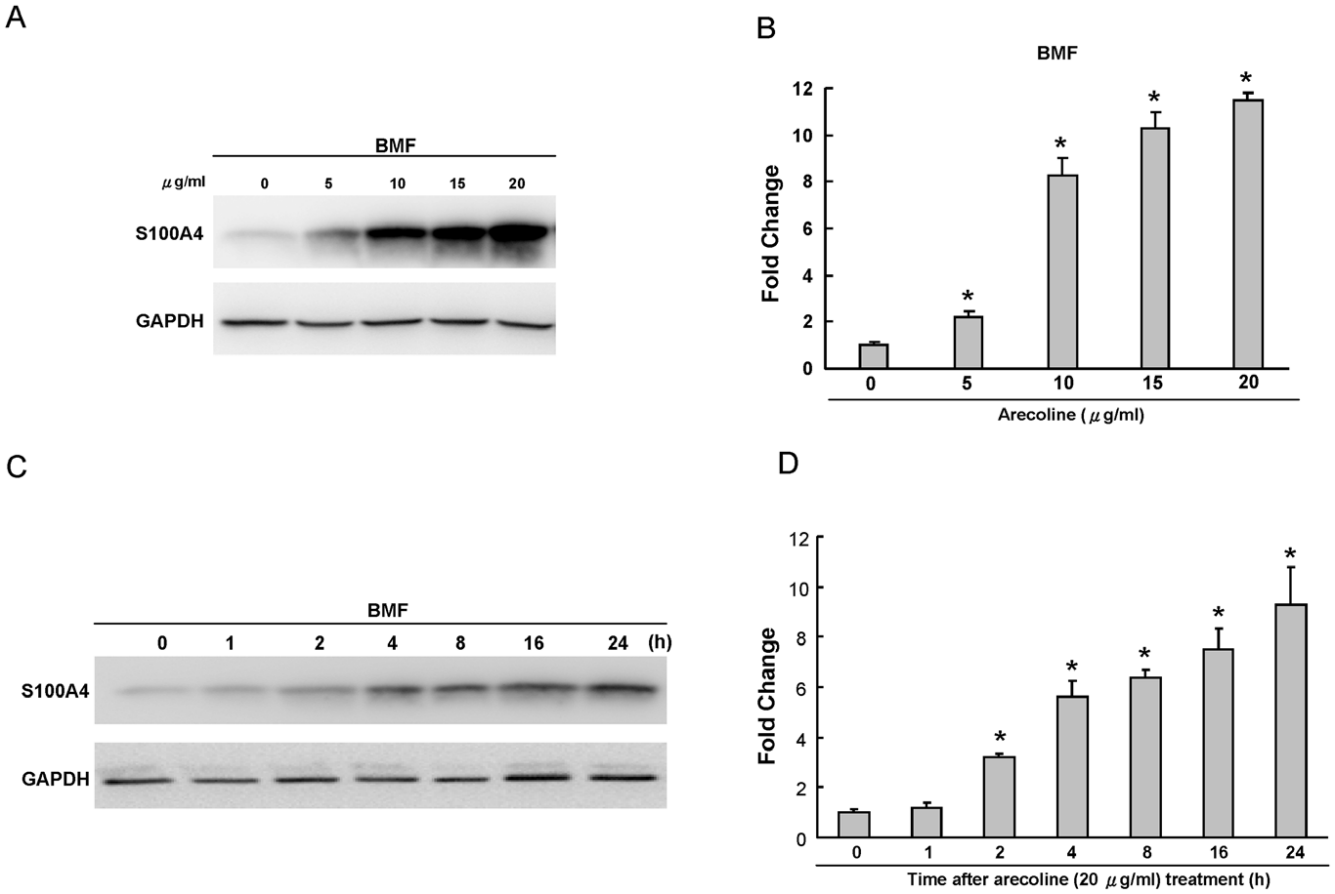
Expression of S100A4 in arecoline-treated BMFs by western blot. (A) BMFs were exposed for 24 h in medium containing various concentrations of arecoline as indicated. GAPDH was performed in order to monitor equal protein loading. (B) Levels of S100A4 protein stimulated by arecoline were measured by densitometer. The relative level of S100A4 protein expression was normalized against GAPDH signal and the control was set as 1.0. Optical density values represent the mean ± SD. * represents significant difference from control values with *p*<0.05. (C) Kinetics of S100A4 expression in BMFs exposed to 20 µg/ml arecoline for 0, 1, 2, 4, 8, 16, and 24 h, respectively. GAPDH was performed in order to monitor equal protein loading. (D) Levels of S100A4 protein stimulated by arecoline were measured by densitometer. The relative level of S100A4 protein expression was normalized against GAPDH signal and the control was set as 1.0. Optical density values represent the mean ± SD. * represents significant difference from control values with *p*<0.05.

To examine the effect of arecoline, the major alkaloid of areca nuts, on the S100A4 expression, two representative BMF strains were treated with arecoline and the levels of protein were measured. The effects of arecoline on the S100A4 expression in two individual cell strains were similar, and their intracellular variations were limited. As shown in figure 2A and B, arecoline was found to upregulate S100A4 protein expression in a dose-dependent manner (p<0.05). In addition, arecoline was also demonstrated to elevate S100A4 expression in a time-dependent manner in BMF cells (Fig. 2C&D, p<0.05).

### NF-κB, ERK, or mTOR Signaling Pathway Involved in Arecoline-induced S100A4 Expression

**Figure 3:**
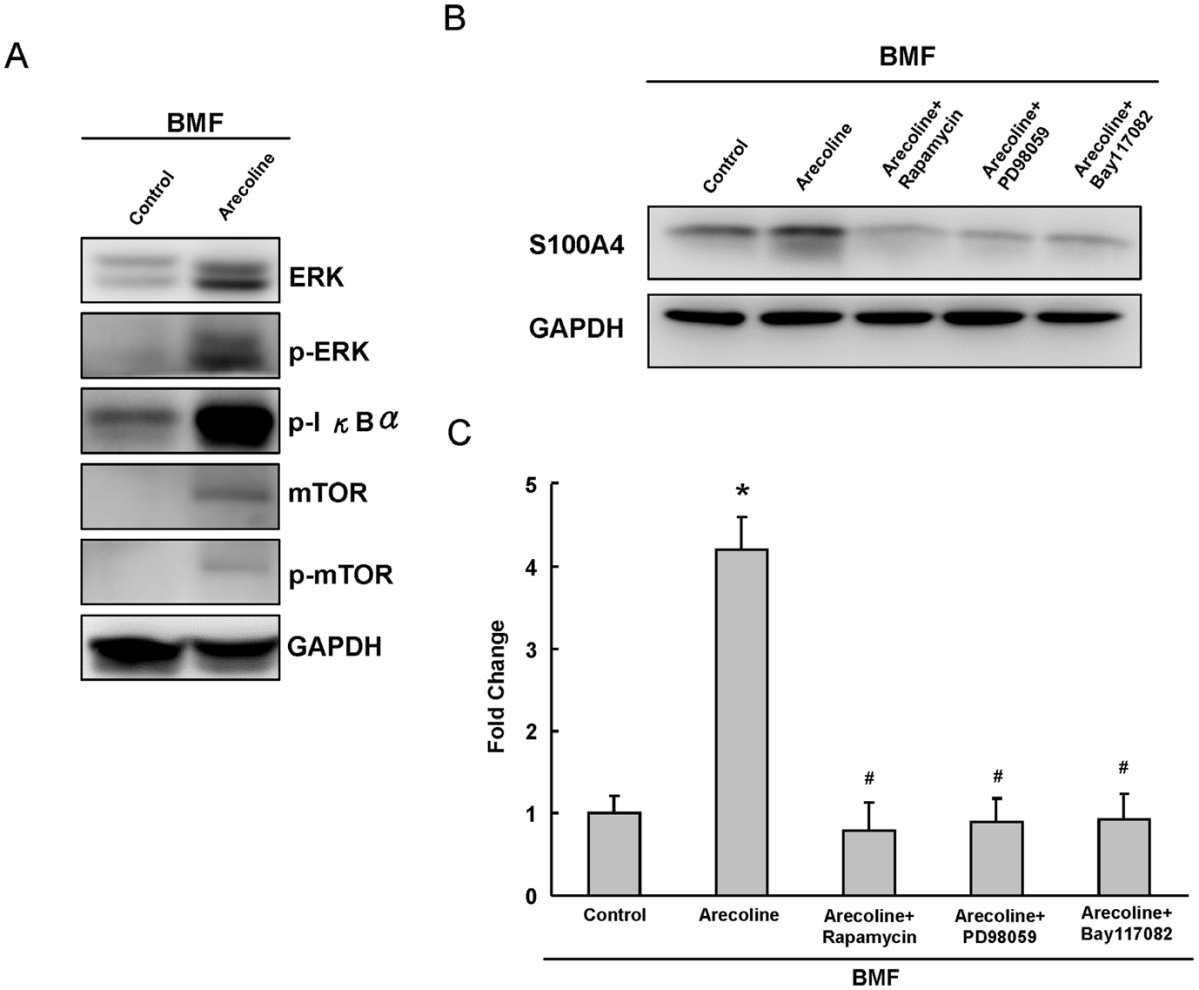
The regulatory effects of rapamycin, PD98059, and Bay117082 on arecoline-induced S100A4 expression in BMFs. (A) Levels of ERK, p-ERK, p-IκBα, mTOR, p-mTOR protein stimulated by 20 µg/ml arecoline were measured by western blotting. GAPDH was performed in order to monitor equal protein loading. (B) BMFs were coincubation with pharmacological agents in the presence of 20 µg/ml arecoline. GAPDH was performed in order to monitor equal protein loading. (C) From the AlphaImager 2000, rapamycin, PD98059, and Bay117082 were found to reduce the arecoline-induced S100A4 expression, respectively. * represents significant difference from control values with p<0.05. ^#^ represents statistically significant values between arecoline alone and arecoline with pharmacological agents; p<0.05.

To further study the possible mechanisms involved in arecoline-induced S100A4 up-regulation, we showed that arecoline treatment increased NF-κB, ERK, or mTOR signaling in BMF cells (Fig. 3A). Rapamycin (mTOR inhibitor), PD98059 (ERK inhibitor), and Bay117082 (NF-κB inhibitor) without cytotoxic concentration were added to search the possible regulatory mechanisms on arecoline-induced S100A4 expression in BMFs. These pharmacological agents were found to inhibit the arecoline-induced S100A4 expression in BMFs (*p*<0.05) (Fig. 3B & Fig. 3C).

### Knockdown of S100A4 Repressed Arecoline-induced Collagen Gel Contraction *via* TIMP1/MMP9 Regulation

**Figure 4:**
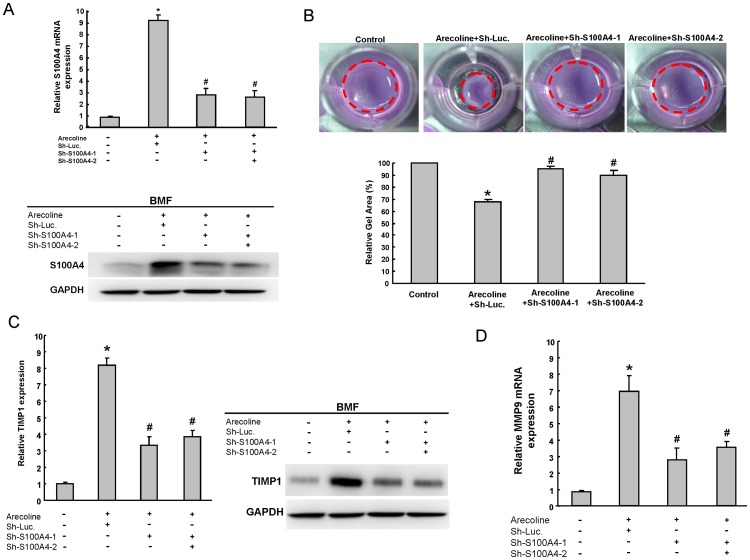
Depletion of S100A4 reversed the phenotype of arecoline-induced collagen gel contraction and TIMP1 expression. (A) The silencing effect of S100A4 shRNA in arecloine-treated BMFs was validated transcriptionally by real-time RT-PCR analysis (*upper panel*). Single cell suspension of BMFs was transduced with sh-Luc or sh-100A4 lentivirus, individually or concurrently, and treated with or without arecoline (20 µg/ml) as indicated. Total proteins prepared from infected cells were prepared and analyzed (*bottom panel*). (B) Single cell suspension of arecoline-treated BMFs infected with S100A4-specific shRNA or control sh-Luc lentivirus was analyzed by collagen gel contraction assay. (C) Real-time RT-PCR analysis (*left panel*) and immunoblotting analysis (*right panel*) of TIMP1 in sh-Luc or S100A4-knockdown BMFs with or without arecoline treatment were analyzed. The amount of GAPDH protein of different crude cell extracts was referred as loading control for further quantification. (D) Real-time RT-PCR analysis of MMP9 in sh-Luc. or S100A4-knockdown BMF cells with or without arecoline treatment were analyzed. *P<0.05 Sh-Luc.+arecoline group versus control group; ^#^P<0.05 Sh-S100A4-1+arecoline or Sh-S100A4-2+ arecoline versus Sh-Luc.+arecoline group.

**Figure 5:**
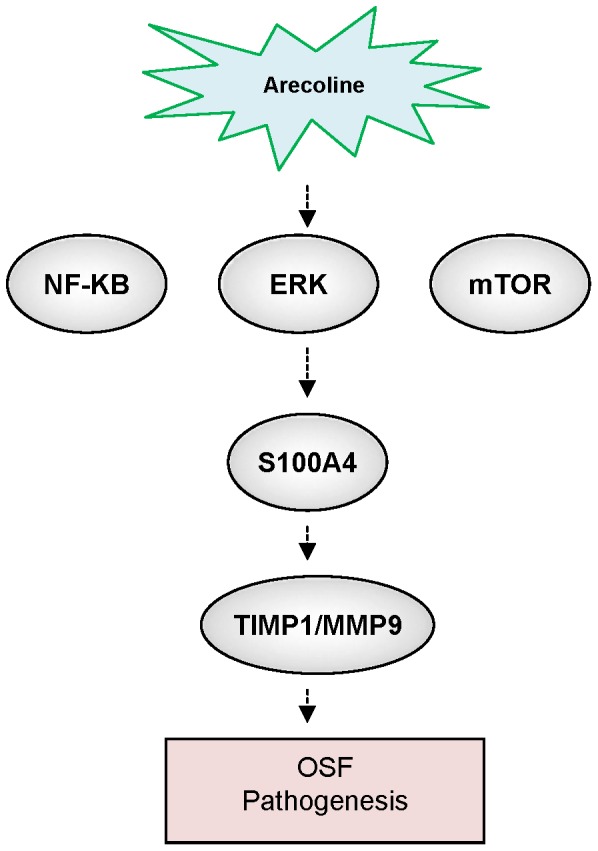
Schematic of the S100A4 signaling pathways promoting the pathogenesis in OSF.

To further investigate whether S100A4 could play a role in maintaining properties of arecoline-treated BMFs, the approach of loss-of-function of S100A4 was first conducted. Down-regulation of S100A4 in arecoline-treated BMFs was achieved by viral transduction with lentiviral vector expressing small hairpin RNA (shRNA) targeting (sh-S100A4-1 and sh-S100A4-2). In addition, lentiviral vector expressing shRNA against luciferase (sh-Luc) was used as control. Real-time RT-PCR and immunoblotting analyses confirmed that lentivirus expressing both sh-S100A4-1 and sh-S100A4 -2 markedly reduced the expression level of arecoline-induced S100A4 transcript and protein expression (Fig. 4A). Knockdown of S100A4 also decreased p-IκB-α expression, but did not change ERK and mTOR expression in arecoline-treated BMFs (Fig. S1). During wound healing and organ fibrosis, myofibroblasts are the major cell type to secret collagen and reorganize the ECM. Deregulation of myofibroblast activity has been found in several organ fibrosis, such as liver, heart, lung, and OSF [17]. The treatment of arecoline significantly induced the contraction of collagen gel-embedded BMFs (Fig. 4B). Importantly, targeting S100A4 abrogated arecoline-induced collagen gel contraction ability in BMFs (Fig. 4C). Previously, we have demonstrated that TIMP1 was upregulated in arecoline-treated BMFs [7]. Notably, S100A4 can promote invasive ability of prostate cancer cells through MMP9 and TIMP1 regulation [18]. However, the possible mechanisms that S100A4 activate the downstream effects including amplification of TIMP1 activity in OSF still remain unclear. S100A4 silencing was demonstrated to reduce the transcripts of protein levels of arecoline-induced TIMP1 and MMP9 expression (Fig. 4C & 4D). In summary, our results suggested that S100A4/TIMP1/MMP9 signaling may play a major switch on the regulation of OSF pathogenesis (Fig. 5).

## Discussion

Based on the epidemiological evidences, OSF is highly associated with the habit of areca quid chewing. However, the exact mechanism of areca nut constituents’ action on the oral mucosal tissue is not fully understood. Herein, we evaluated the role of S100A4 in the pathogenesis of OSF. We treated BMFs with arecoline to examine its influence on S100A4 to search for the possible pathogenesis of areca quid-associated OSF both in vitro and in vivo. To the best of our knowledge, we first found that arecoline is capable of stimulating S100A4 expression in human BMFs. This suggests that one of the pathogenetic mechanisms of OSF may be the synthesis of S100A4 expression. Additionally, pre-treatment with pharmacologic agents markedly inhibited the arecoline-induced S100A4 expression. Depletion of S100A4 by lentiviral-mediated knockdown reversed arecoline-induced TIMP1 and MMP9 expression of arecoline-stimulated fibroblasts (Fig. 4). Our data first demonstrated the crucial role of S100A4 in the balance ratio of MMP9 to TIMP1, leading to synthesis and deposition of ECM components in OSF.

EMT (epithelial-mesenchymal transition), a de-differentiation program converting adherent epithelial cells into individual migratory cells, is critical for the embryonic development, oncogenic progression of tumor cells, and fibrosis [19], [20]. Enhanced EMT characteristic is associated with the development of renal fibrosis [21]. Local fibroblasts of tissues are considered as the predominant source of myofibroblasts, but myofibroblasts could also come from other cell types within tissues, such as epithelial cells, endothelial cells, and hepatocytes, through EMT process [22]. The typical molecular feature of differentiated myofibroblasts is the expression of alpha smooth muscle actin (α-SMA) and fibronectin [22]. From our previous studies, the upregulation of several EMT-related molecules, such as PAI-1 [8], IGF-1 [23], and NF-κB [11], are observed in OSF. Arecoline, the major alkaloid in areca nut, could induce the expression of vimentin [5] and IL-6 [9] in human BMFs. These data suggest the potential involvement of EMT program in the pathogenesis of OSF. S100A4, a member of CBPs, is directly controlled by Wnt/ß-catenin signaling pathway as a master mediator in EMT [24]. S100A4 is involved in a variety of biological effects including cell motility, survival, differentiation, and cytoskeletal organization [25], [26]. However, the detailed molecular mechanisms involved in the regulatory links between S100A4-mediated EMT and myofibroblast in OSF are still poorly understood. Therefore, better understanding of the biological characteristics of EMT axis in OSF will provide us with new therapeutic approaches. Further research effort is needed in this area.

The ERK cascade is a central signaling pathway that participates in the regulation of proliferation phenotypes. In this study, PD98059 was found to reduce S100A4 protein expression by arecoline in BMFs. Arecoline and areca nut extracts have been shown to regulate the expression of several genes dependent on the activation of MAPKs and NF-κB in human oral keratinocytes and fibroblasts [27]. Consistently, Eugene et al. [28] have demonstrated NF-κB-binding site within enhancer element located within the first intron of S100A4 gene. These data support our findings that the activation of the ERK/NF-κB signaling may be involved in arecoline-induced S100A4 expression in BMFs. To the best of our knowledge, our study first demonstrated that S100A4 highly expressed in OSF tissues and arecoline was capable of stimulating S100A4 expression in BMFs. Furthermore, arecoline-induced S100A4 expression could be blocked by co-treatment of BMFs with Rapamycin, PD98059, and Bay117082. Moreover, knockdown of S100A4 could reverse the arecoline-induced collogen gel contraction. Overall, our present research showed that S100A4/TIMP1/MMP9 could play a major role in the molecular pathogenesis of OSF. Targeting S100A4/TIMP1/MMP9 signaling might be a potential therapeutic target for OSF.

## Supporting Information

Figure S1 **NF-κB, ERK, or mTOR involved in arecoline-induced S100A4 expression.** Immunoblotting analysis of ERK, p-ERK, mTOR, p-mTOR, and p-IκBα expression in sh-Luc or S100A4-knockdown BMFs with or without arecoline treatment were analyzed. The amount of GAPDH protein of different crude cell extracts was referred as loading control for further quantification.
(TIFF)
