# Supplementary figures and images for: Elevation of S100A4 Expression in Buccal Mucosal Fibroblasts by Arecoline: Involvement in the Pathogenesis of Oral Submucous Fibrosis
Source: PLoS One. 2013 Jan 31;8(1):e55122. doi: 10.1371/journal.pone.0055122 (PMC3561403; doi:10.1371/journal.pone.0055122)

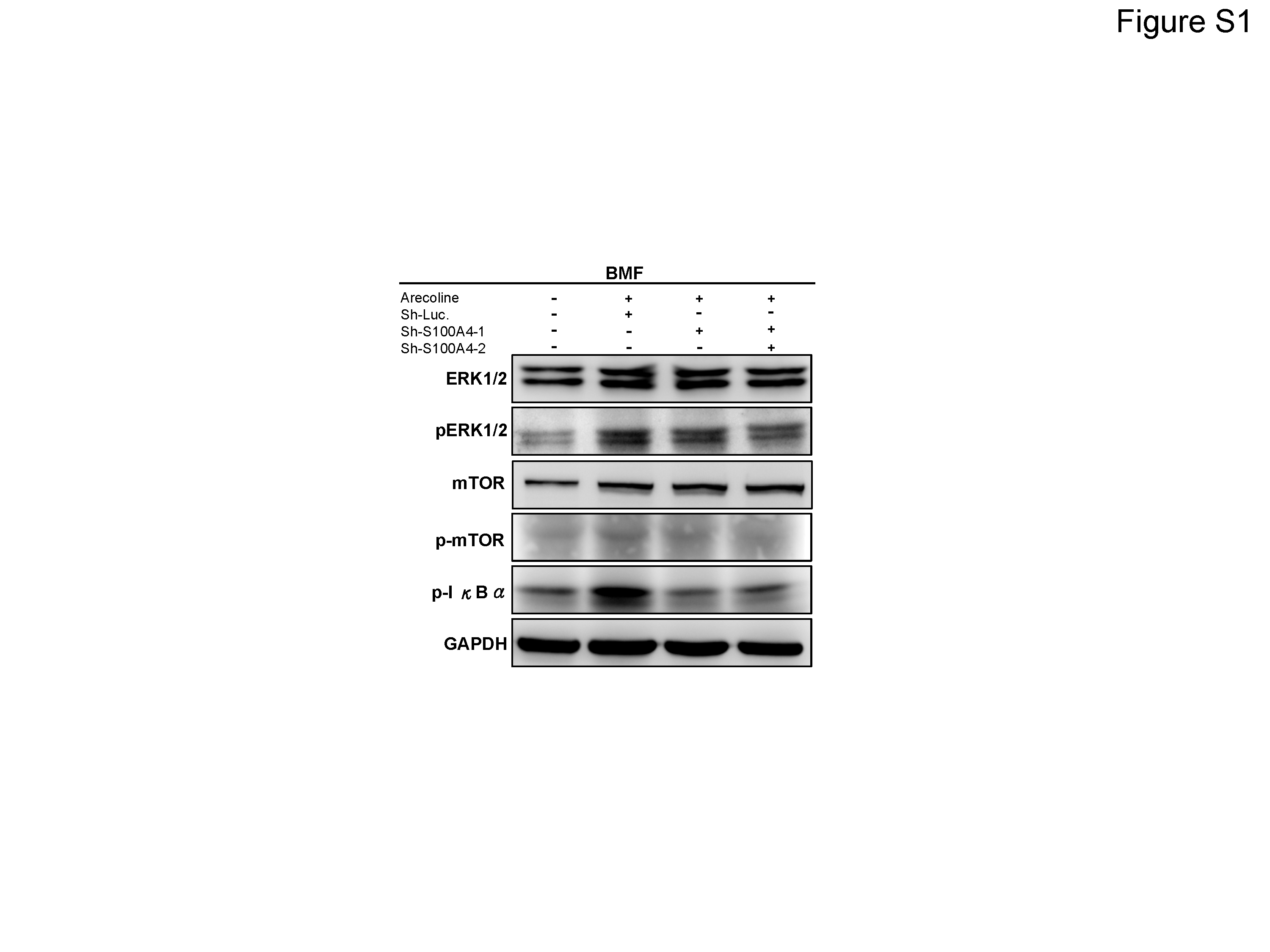

Supplement: Figure S1 — NF-κB, ERK, or mTOR involved in arecoline-induced S100A4 expression. Immunoblotting analysis of ERK, p-ERK, mTOR, p-mTOR, and p-IκBα expression in sh-Luc or S100A4-knockdown BMFs with or without arecoline treatment were analyzed. The amount of GAPDH protein of different crude cell extracts was referred as loading control for further quantification. (TIFF) [file pone.0055122.s001.tiff]
